# Finite Element Analysis and Fatigue Test of INTEGRA Dental Implant System

**DOI:** 10.3390/ma17051213

**Published:** 2024-03-06

**Authors:** Rafał Zieliński, Sebastian Lipa, Martyna Piechaczek, Jerzy Sowiński, Agata Kołkowska, Wojciech Simka

**Affiliations:** 1 Stomatologia na Ksiezym Mlynie, 16 D Tymienieckiego, 90-365 Lodz, Poland; martyna.piechaczek2001@gmail.com; 2 Institute of Materials Science and Engineering, Lodz University of Technology, 90-924 Lodz, Poland; sebastian.lipa@p.lodz.pl; 3 Private Dental Clinic, Tetmajera 3A Rd., 05-080 Izabelin C, Poland; jersow@gmail.com; 4 Chemistry Students Research Society, Faculty of Chemistry, Silesian University of Technology, 44-100 Gliwice, Poland; agatkol653@student.polsl.pl; 5 Department of Inorganic Chemistry, Faculty of Chemistry, Analytical Chemistry and Electrochemistry, Silesian University of Technology, 44-100 Gliwice, Poland

**Keywords:** INTEGRA IMPLANTS, finite element analysis, PN-EN 14801, INTEGRA OPTIMA, dental implant cracks, dental implant damage

## Abstract

The study involved numerical FEA (finite element analysis) of dental implants. Based on this, fatigue tests were conducted according to the PN-EN 14801 standard required for the certification of dental products. Thanks to the research methodology developed by the authors, it was possible to conduct a thorough analysis of the impact of external and internal factors such as material, geometry, loading, and assembly of the dental system on the achieved value of fatigue strength limit in the examined object. For this purpose, FEM studies were based on identifying potential sites of fatigue crack initiation in reference to the results of the test conducted on a real model. The actions described in the study helped in the final evaluation of the dental system design process named by the manufacturer as INTEGRA OPTIMA 3.35. The objective of the research was to identify potential sites for fatigue crack initiation in a selected dental system built on the INTEGRA OPTIMA 3.35 set. The material used in the research was titanium grade 4. A map of reduced von Mises stresses was used to search for potential fatigue crack areas. The research [loading] was conducted on two mutually perpendicular planes positioned in such a way that the edge intersecting the planes coincided with the axis of the system. The research indicated that the connecting screw showed the least sensitivity (stress change) to the change in the loading plane, while the value of preload has a significant impact on the achieved fatigue strength of the system. In contrast, the endosteal implant (root) and the prosthetic connector showed the greatest sensitivity to the change in the loading plane. The method of mounting [securing] the endosteal implant using a holder, despite meeting the standards, may contribute to generating excessive stress concentration in the threaded part. Observation of the prosthetic connector in the Optima 3.35 system, cyclically loaded with a force of F ≈ 300 N in the area of the upper hexagonal peg, revealed a fatigue fracture. The observed change in stress peak in the dental connector for two different force application surfaces shows that the positioning of the dental system (setting of the socket in relation to the force action plane) is significantly decisive in estimating the limited fatigue strength.

## 1. Introduction

Implants, implant-supported single crowns, and fixed dental prostheses boast impressive survival rates of 97–98% over five years [1], establishing them as dependable options for replacing missing teeth. However, these treatments are not without potential complications, which can be mechanical, technical, biological, or aesthetic in nature [2,3]. Successful implant dentistry can be directly related to the quality and quantity of bone at the recipient site of the implant. Over the years, bone grafts have been used for the treatment of various osseous defects. Due to the widespread acceptance of dental implants, interest in bone reconstruction for the oral cavity has increased dramatically over the past decade. Many patients who request implant rehabilitation require ancillary procedures to increase the quantity and quality of the recipient’s bone [4].

In some clinical situations, such as in horizontal bone augmentation, the tenting screw technique in the posterior mandible is used [5]. Alveolar ridge preservation (ARP) is another method of decreasing bone resorption following tooth extraction and facilitating prosthetically driven implant placement [6].

The primary goal for clinicians is to minimize or prevent factors that could lead to failure, such as component fracture or progressive bone loss. These contributing factors are broadly divided into patient-dependent factors (like hygiene, peri-implant microbiota, and bone quality) and professional-dependent factors (including implant selection, restoration design, occlusal adjustment, and others that may increase the load on the implant–restoration complex). The culmination of these biomechanical factors leads to stress on the prostheses, prosthetic components (such as screws and abutments), implants, and the surrounding bone. If not adequately managed, this stress can exceed the implant material’s breaking strain, causing fracture or leading to pathological overload, potentially resulting in bone loss if resorption outweighs bone formation [7,8].

Although randomized controlled clinical trials are ideal for assessing the impact of these biomechanical factors, ethical, deontological, and economic challenges can impede their execution. In vitro test methods, however, offer a way to recreate various biomechanical conditions to study their effects on implants, peri-implant bone, and the implant–prosthesis complex.

To evaluate outcomes based on load conditions and other experimental characteristics, common in vitro methods include numerical simulation analysis through finite elements, validated by techniques such as extensometry, photoelasticity, photogrammetry, fractal analysis, and, more recently, digital image correlation (DIC) [9,10,11,12,13]. Despite this, experimental verification through simulation is crucial to ascertain the long-term durability of components under real loads. Therefore, conducting fatigue tests under realistic loading conditions and effective chewing scenarios is essential for accurate evaluation.

The study involved numerical FEA (finite element analysis) of dental implants. The FEM studies were correlated with the fatigue test according to the PN-EN 14801 (Dentistry—Implants—Dynamic Fatigue Test for Endosseous Dental Implants) standard [14] titled: Fatigue Testing of Endosseous Dental Implants. The evaluation of the numerical model/simulation was based on the analysis of the distribution/maps of reduced von Mises stresses, which revealed phenomena associated with triaxial stress states, effects of screw tightening, and the impact of asymmetric loading due to the random position of the hexagonal socket in relation to the base/bone and the key socket in the connector screw head on the possible result of the assembly’s fatigue strength. The research was treated as a qualitative analysis, and the results aided in interpreting the fatigue mechanism of the tested object. The research was conducted using the Ansys Workbench 2020 environment. The objective of the research was to identify potential sites for fatigue crack initiation in a selected dental system built on the INTEGRA OPTIMA 3.35 implant series, which were subjected to fatigue tests.

## 2. Materials and Methods

### 2.1. Description of the Set

**Figure 1:**
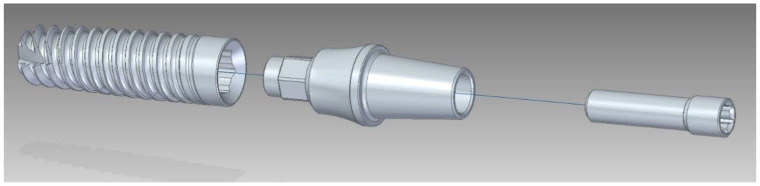
Geometrical model of the OPTIMA 3.35 dental set.

The system (set) INTEGRA OPTIMA 3.35 (INTEGRA IMPLANTS; Rd. Dostawcza 14; Lodz, Poland), used in the study, belonged to the type of implantprostheses, where the primary component was the so-called endosteal implant (internal bone implant) with an external thread holding it in the jawbone tissue. This implant was connected to the prosthetic connector (supra-bone part), upon which the tooth prosthesis (crown) was reconstructed during the implantological procedure. The entire system, implant + connector, was connected by a connector screw (Figure 1). The tightening torque of the connecting screw was set to M = 20 Ncm. It was checked each time using a torque wrench. To eliminate rotational movement between the system elements, a hexagonal socket was introduced in the endosteal implant, and a corresponding hexagonal-cylindrical peg was introduced in the prosthetic connector. Additionally, the prosthetic connector elements were seated on the conical surface of the internal bone implant. From the perspective of implantation and numerical modeling, a characteristic feature of the adopted system was ensuring the coaxial alignment of all elements relative to each other. The fifteen samples were accepted for testing, of which three samples were subjected to static tests to determine the stress level. The material was characterized according to the specifications provided by the manufacturer—grade 4 [ISO 5832-2:2018] (Implants for surgery—Metallic materials—Part 2: Unalloyed titanium) [15] (Table 1).

A bilinear isotropic model with hardening after yielding was used for the calculations, defining the elastic and plastic zones and giving the material a nonlinear working character.

**Figure 2:**
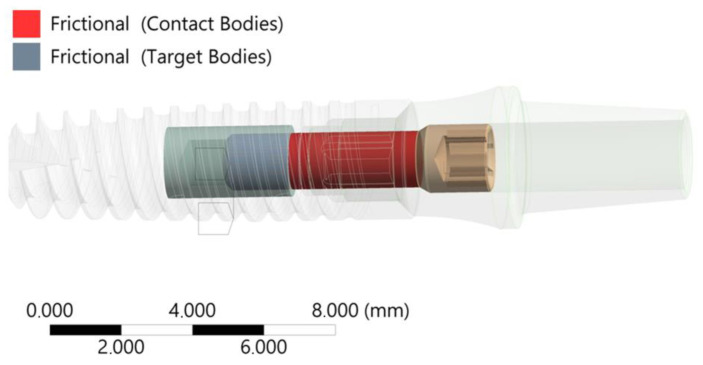
Contact condition screw—implant (thread domain)—friction coefficient in the thread µ = 0.4.

The 3D geometric model used for the calculations, consisting of the endosteal implant, connector screw, and abutment, was further divided into a domain in the area of the connector screw thread with the internal bone implant (Figure 1 and Figure 2). A 3.35-diameter dental implant was chosen because it is the smallest diameter available in the INTEGRA OPTIMA dental implant system range.

### 2.2. FEA of the INTEGRA OPTIMA 3.35

**Figure 3:**
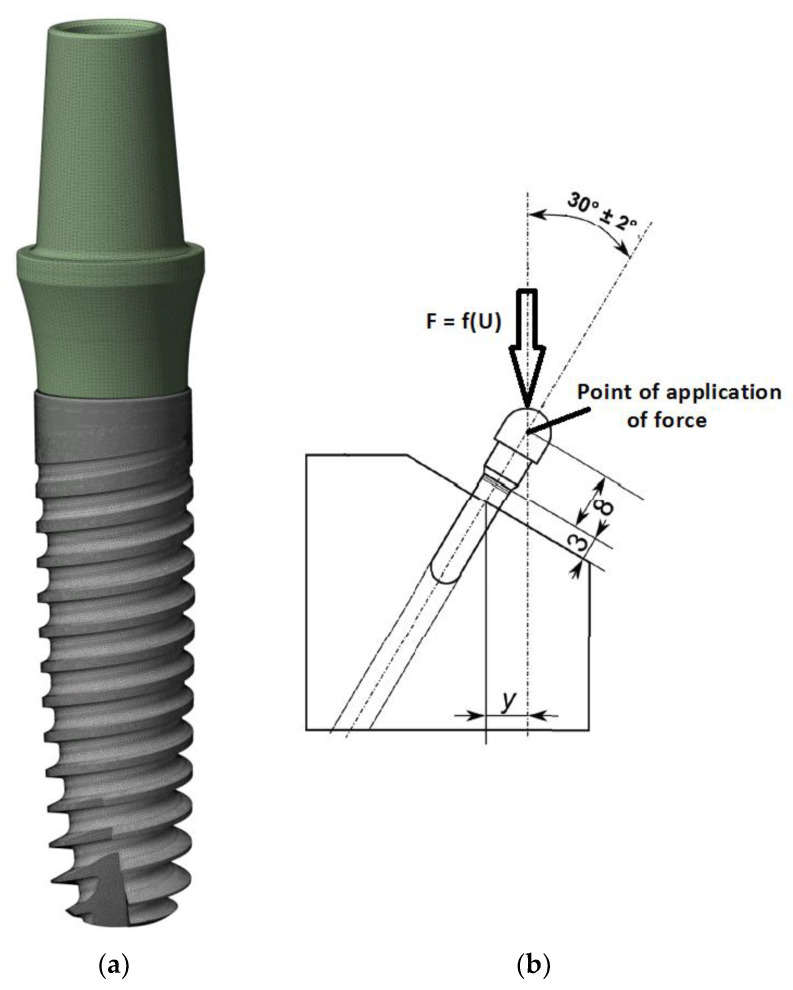
(**a**) FEA mesh of the entire set with division into elements and (**b**) boundary conditions—point of force application.

The “Bolt Thread” contact tool was used to model the helical line of the connection. The FEA discrete model was marked with 2.933.798 elements and 4.900.775 nodes, resulting in a satisfactory mesh quality confirmed by the “Element Quality” metric EQ = 0.696. Solid 187, Conta 174, and Targe 170 elements were used for the calculations (Figure 3).

FEA calculations were conducted in two “loading” planes, X-Z and X-Y, examining the effect of the random position of the hexagonal socket of the endosteal implant relative to the load force on the fatigue mechanism of the system. The boundary conditions of the FEA model were defined to reflect loading situations on the testing machine, as described in the standard PN-EN 14801 (Figure 2 and Figure 3). A fixed support was selected to define the area of the implant-bone connection. This support took away six degrees of freedom at each node in the mesh. Bearing in mind that the purpose of the selected support was to reflect the state of osteointegration of the implant with the bone, the adopted support should be treated as a simplification [large implant stiffening] of real conditions, meeting the provisions of the standard where this support has a defined stiffness higher than 3 GPa. In the analysis, the direct, actual distribution of the patient’s chewing forces was omitted; the implant loading was referred to the requirements of the standard in order to correlate both studies with each other. The load was applied by gradually changing the displacement at the point of force application. The load force was read from a fixed support marked in the area of the studied implant. Nonlinear geometric deformation conditions were adopted in the analyzed FEA model.

To complement the FEA model, a friction coefficient between the screw thread and the implant was introduced at µ = 0.4 (Figure 3). Additionally, the preload tension of the connecting screw resulting from the moment of tightening the connector with the implant, moment M = 20 Ncm, was modeled.

To search for potential fatigue crack areas, the analysis of the most stressed fields was used. For this purpose, a map of reduced von Mises stresses was used. A gradual increase in the load on the set was assumed, and the time step at which the ‘entry’ of each element of the system into the area of permanent deformation was observed was marked. Based on this, the potential sources of cracks were assumed to be the places with the maximum value of reduced von Mises stresses close to the yield limit in the applied material model.

### 2.3. Fatigue Strength Testing

#### 2.3.1. Description of Wöhler Curve

Good practice in designing and evaluating new dental systems is to use available, proven research standards that aim to replicate the most adverse working condition of the object. One of them is the PN-EN 14801 standard describing the fatigue durability test. In dental research, fatigue studies are conducted using models based on stress, strain, and fracture mechanics. The most commonly used method in this field involves stress-based models, employing S-N curves and the staircase method. These methods provide fundamental information about fatigue behavior and help predict the overall lifespan of dental materials.

The S-N curve, also known as the Wöhler curve, graphically represents the number of cycles a specimen can withstand before fracturing under varying cyclic load ranges. This load range is defined by a set upper and lower limit, which remains constant during testing. On the S-N curve, the *y*-axis typically represents the force range in Newtons or, more commonly, the stress range in N/mm^2^, either in a linear or logarithmic scale. The *x*-axis displays the number of applied cycles, always on a logarithmic scale. These curves show that higher loads lead to fracture at a lower number of cycles, while lower loads can endure more cycles before failing. The S-N curve is advantageous as it can be interpreted as an accelerated life testing, allowing for the assessment of component response over its lifetime (number of cycles at a specific frequency) to various load levels. It also suggests the existence of a fatigue limit. However, this model often requires testing a large number of specimens at different load levels, which can be time-consuming and costly. This makes it a less practical approach when only limited data is available. The evaluation of fatigue results typically uses probabilistic criteria, leading to the concept of the S-N-P field.

The fatigue limit is a key concept in these studies, referring to the maximum load level a material can endure without fracturing for an indefinite number of load cycles. Understanding and accurately determining this limit is crucial for predicting the longevity and reliability of dental materials under repetitive stress.

#### 2.3.2. Fatigue Test of the INTEGRA OPTIMA 3.35

**Figure 4:**
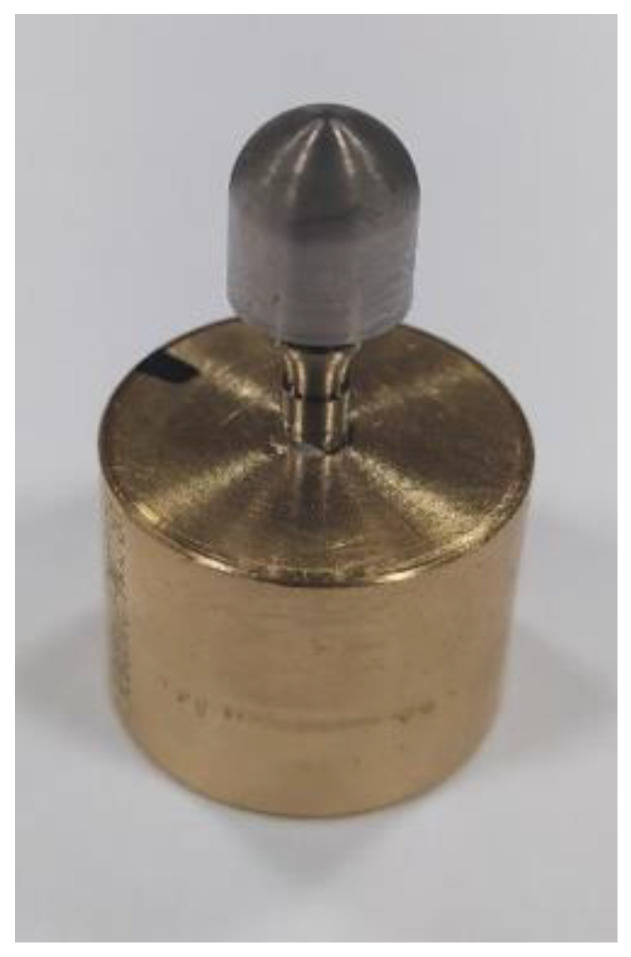
The tested system OPTIMA 3.35 on a brass base.

**Figure 5:**
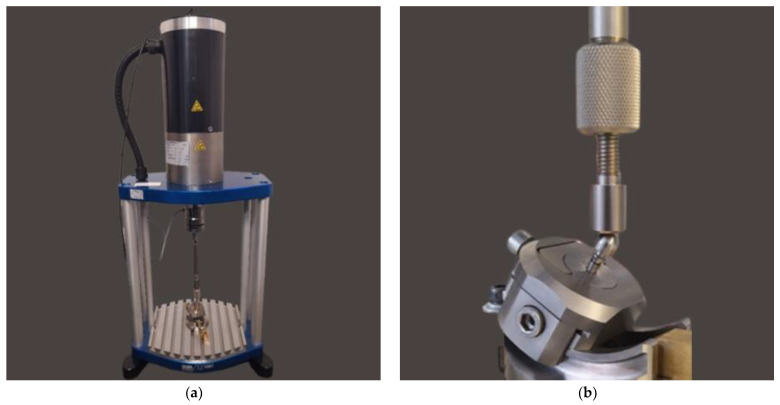
(**a**) Research Workstation: Dora 14801. (**b**) The most favorable mounting of the system to the holder at the testing station met the guidelines of PN-EN 14801.

For fatigue strength and life cycle testing at selected load levels, a dental system consisting of an endosteal implant, connector screw, and prosthetic connector of the OPTIMA 3.35 series was selected (Figure 1 and Figure 4). Fatigue tests of dental implants were conducted in accordance with the requirements contained in the standard PN-EN 14801 titled Fatigue Testing of Endosseous Dental Implants. The tests were carried out using a setup on the Dora 14801 strength testing machine from Larado Prüf- & Mess-Technik GmbH (Bingen am Rhein, Germany) (Figure 5a).

The test was conducted at a temperature of 21 ± 5 °C and under unilateral negative sinusoidal loading. Cyclic loads in the unrestricted area were selected using a stepwise method. The maximum load level was estimated in the range of 40–70% of the force value corresponding to the emergency strength of the dental system. Fatigue strength tests and the determination of the Wöhler curve for Optima 3.35 systems were conducted at a frequency of 10 Hz. The number of cycles assumed to determine the fatigue strength of the set is 5 × 10^6^. The system was assembled by tightening torque wrenching the screw with a torque of M = 20 Ncm, which corresponded to the input parameters used in the numerical studies. The Optima 3.35 system was mounted in a calibrated hole in a brass cylindrical base ø = 20 mm using acrylic. This method ensured the most favorable mounting of the system to the holder at the testing station and met the guidelines of PN-EN 14801 (Figure 5b), where the support is intended to provide stiffness higher than 3 GPa. The positioning of the dental system, i.e., its positioning at the testing station, was carried out in such a way as to meet the provisions of the standard PN-EN 14801, as well as the conditions adopted in the FEA numerical analysis. The test was conducted on 18 samples, with three samples subjected to static tests to determine the cyclic force that would destroy the system. The results of three samples were discarded as statistically insignificant [two samples tested at the level of F ≈ 150 N underwent n = 5 × 10^5^ cycles; one sample loaded with a force of F ≈ 300–320 N experienced a sudden loss of system mounting stiffness.

The analysis of the results from the conducted fatigue tests was supplemented with SEM microscope images at various magnifications. For this purpose, the Jeol JSM-6610 (Tokyo, Japan) device was used, operating in the secondary electron (SE) mode at an accelerating voltage of 20 kV. The analysis results were also correlated with the maps of reduced von Mises stresses obtained through FEA analysis.

### 2.4. Hypothesis Zero

The unlimited fatigue strength of the Optima 3.35 system is influenced by factors such as load force level, pre-tension of the connecting screw, and method of mounting the implant with the base. The observed fatigue fractures in the prosthetic connector, especially in the area of the upper hexagonal peg, suggest potential sites for the development of cracks and propagation of fatigue foci. The positioning of the dental system, specifically the setting of the socket in relation to the force action plane, significantly impacts the estimated limited fatigue strength. The method of modeling, correlated with fatigue testing, not only elucidates the impact of the system’s geometry on test results but also provides insights into loading-induced phenomena that play a crucial role in estimating the unlimited fatigue strength of the system.

## 3. Results

### 3.1. Results of FEA of INTEGRA OPTIMA 3.35

**Figure 6:**
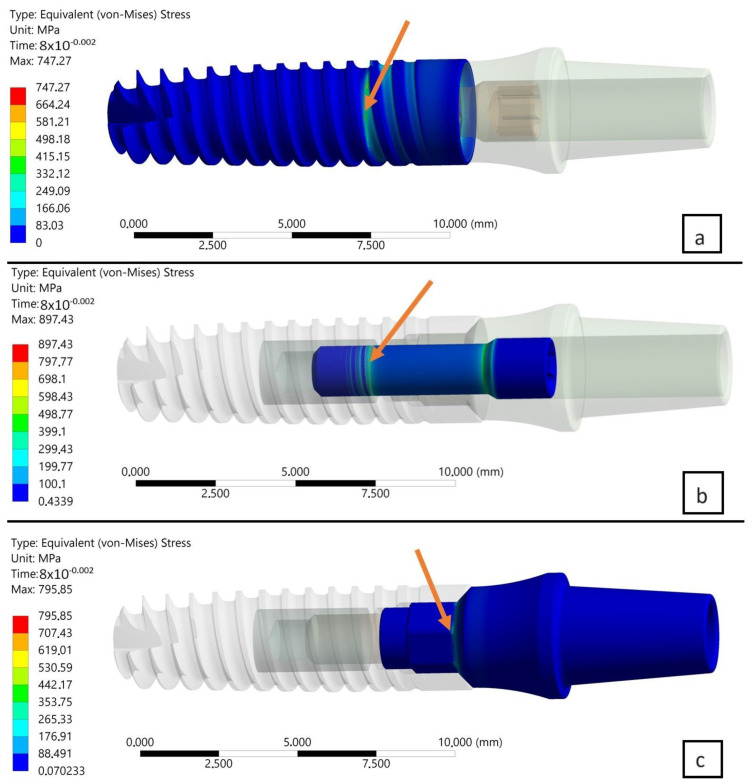
(**a**) Map of reduced stresses of the endosteal implant for T = 0.08. (**b**) Map of reduced stresses of the connecting screw joining the endosteal implant with the prosthetic connector for T = 0.08. (**c**) Map of reduced stresses of the prosthetic connector for T = 0.08. Arrow shows the highest stresses.

**Figure 7:**
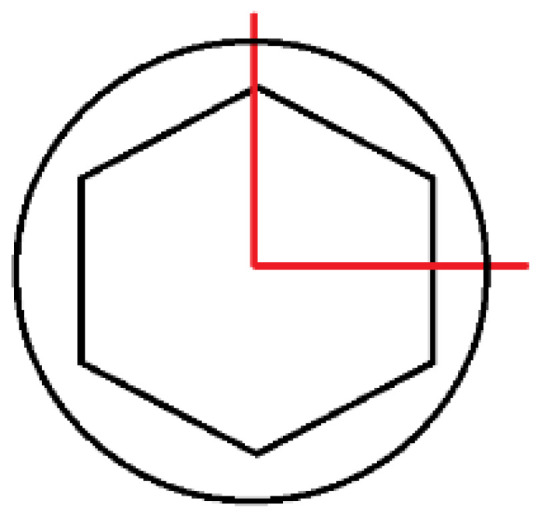
Geometry of the hexagonal peg and marked [in red] planes of load action.

**Figure 8:**
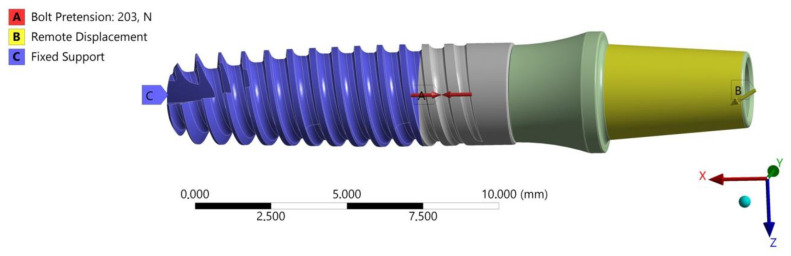
Boundary conditions for the OPTIMA 3.35 dental set; loading in the X-Z plane.

The research and analysis conducted identified potential sources of fatigue cracking for the INTEGRA OPTIMA 3.35 series of endosteal dental implants (Figure 6, Figure 7 and Figure 8). Utilizing the adopted research methodology, three areas were identified as influencing the course of cyclic loading and, consequently, the fatigue strength, indicated with an arrow:

- The area of the external thread of the endosteal implant at its “exit” from the fixed support reflecting the assembly’s anchoring in the substrate (jawbone or maxilla).
- The area of the screw thread corresponding to the position where the connector screw exits the implant.
- The area of the upper part of the hexagonal-cylindrical peg of the prosthetic connector.

In the study, deviations in the values of reduced stresses were observed as a result of changing the load plane. Two planes of load were selected and positioned perpendicularly to each other. The edge of the intersection of both planes was placed along the axis of the set perpendicular to the frontal surface of the implant. The positions of the chosen planes of load were identical to the two different planes of symmetry of the hexagonal socket of the implant used.The degree of deviation was examined in two selected computational steps, i.e., T = 0.08 s and T = 0.1 s (Table 2). For the analyzed numerical model, this corresponded to the “entry” of the assembly [parts/structure] into a state of permanent deformation. Consequently, from the comparison of maximum peaks of reduced von Mises stresses, the following was found:

- Comparing the two planes of load with each other, i.e., X-Z and X-Y, in subsequent time steps, a decrease in stress was observed in the area of the implant by, respectively, 6% (0.08 s) and 5.3% (0.1 s).
- Comparing the two planes of load with each other, i.e., X-Z and X-Y, in subsequent time steps, an increase in stress was observed in the area of the connecting screw by, respectively, 1.3% (0.08 s) and 1.9% (0.1 s).
- Comparing the two planes of load with each other, i.e., X-Z and X-Y, in subsequent time steps, an increase in stress was observed in the area of the connector by, respectively, 4.4% (0.08 s) and 5.5% (0.1 s).

**Figure 9:**
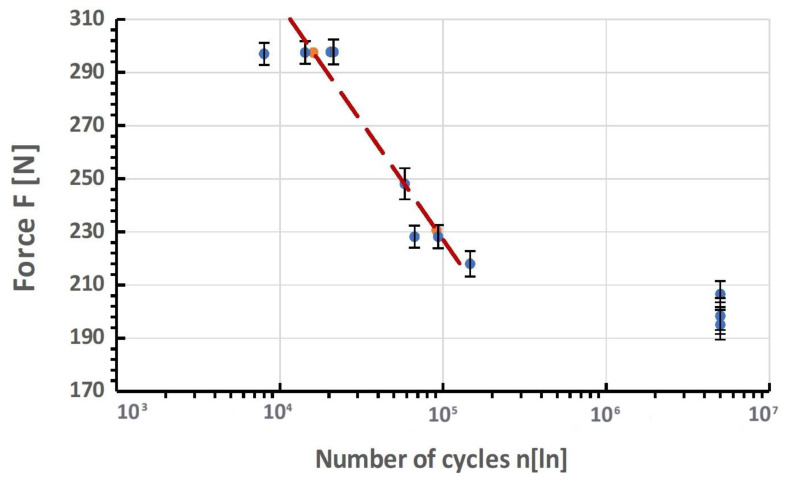
Number of stress cycles (S-N curve) or Wöhler curve for the Optima 3.35 dental system (material: titanium).

Further observations from the research indicated that the connecting screw showed the least sensitivity (stress change) to the change in the loading plane. In contrast, the endosteal implant (root) and the prosthetic connector showed the greatest sensitivity to the change in the loading plane. The cause of this condition was attributed to the following:

- The change in the loaded cross-sectional area resulting from a different position of the hexagonal-cylindrical peg of the connector relative to the action of the loading plane (Figure 7).
- The stiffness of the support fixing the implant, the greater it is, the greater the increase in stress concentration in the area where the implant exits the fixation area [holder, bone] (Figure 9).

As a result of subsequent analysis, it was determined that the outcome of the numerical test varied when the same loading plane was applied but at different computational steps, specifically T = 0.08 s and T = 0.1 s, for individually analyzed objects. The most significant increase in the peak of reduced von Mises stresses was noted in the area of the implant, about 12.5–13%, indicating that this element was just entering the area of permanent deformation. A different level of stress was noted in other parts of the assembly, namely the connector screw and the abutment. A maximum of reduced stresses slightly higher than the accepted yield limit of the material was observed in the area of the screw. This condition was attributed to the initial tension of the screw resulting from the torque applied during the assembly. Analyzing the load force value for this particular moment, it was determined that this state corresponded to the reaction force in the entire dental system at T = 0.08, s–F = 126 N, whereas at T = 0.1, s–F = 183 N.

### 3.2. Results of Fatigue Tests

**Figure 10:**
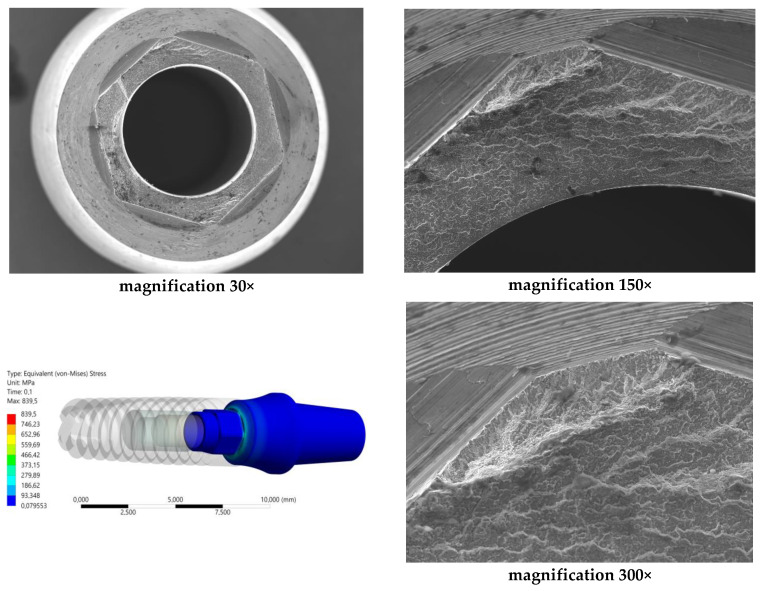
Identification of the most stressed areas in the connector and SEM microscope study results; visible: fatigue focus observed in the upper area of the fracture—corner, perifocal area, primary displacements, and fatigue lines. Load force F ≈ 300 N.

**Figure 11:**
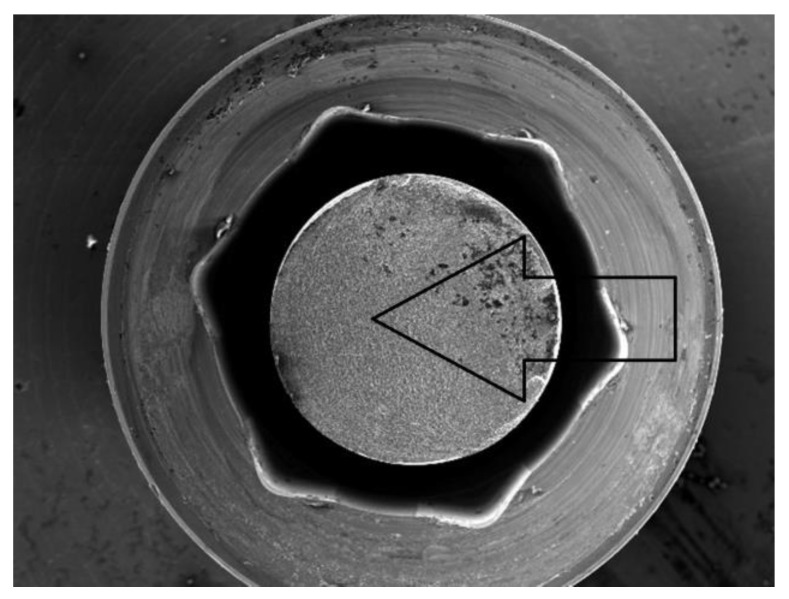
SEM Image (30×) of the breakthrough in the area of the connecting screw under the load force F ≈ 300 N (plastic fracture).

**Figure 12:**
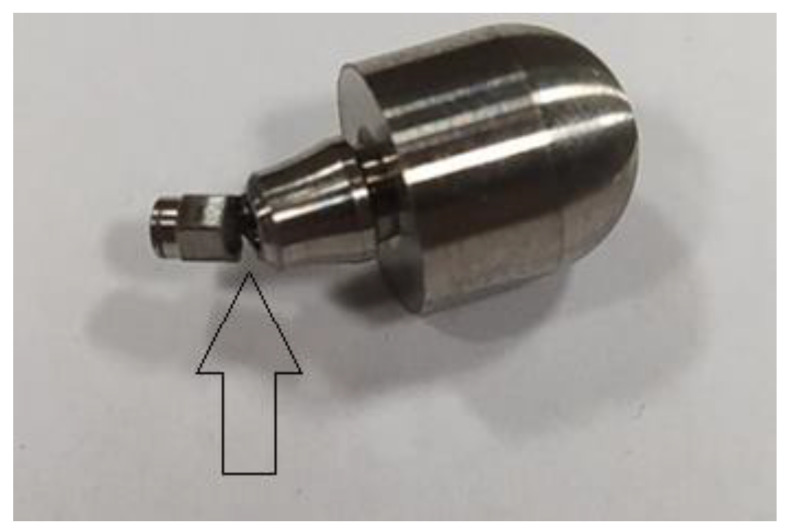
Macro photo of a broken hexagonal peg of the prosthetic connector.

Before commencing the tests, no significant material and technological defects were observed on the surface of the tested systems that would significantly affect the test results (deviations from geometrical features, discolorations, deposits, and damage marks). The torque of the system was checked each time in each sample with a torque wrench. The results for the selected dental system are presented in Table 3 and Figure 10, Figure 11 and Figure 12. Based on the results presented in the tables below, load force as a function of the number of life cycles, a Wöhler fatigue curve was plotted on logarithmic axes corresponding to the number of cycles. Standard deviations shown on the charts referred to deviations in loadforce values during the test for one level of loading. On the constructed Wöhler curve, three characteristic levels of loads were marked: F1 ≈ 200 N, F2 ≈ 220–250 N, and F1 ≈ 300 N. The fatigue strength of the dental system was determined at a level of F = 200–210 N, which corresponded to a number of cycles n = 5 × 10^6^. Fatigue fractures were mainly observed in the upper area of the hexagonal slot of the prosthetic connector. In the SEM images in the area of the fatigue fracture, numerous fatigue foci were observed, mostly running from the edges/external surfaces of the construction, concentrated in the corner. In this field, characteristic perifocal areas, primary displacements, and lines of stage-wise crack growth were also visible. Additionally, plastic fractures in the area of the connecting screw were observed in the SEM images.

## 4. Discussion

### 4.1. Fatigue Test

For the initial and final stages of the development of new dental systems, it is advisable to conduct tests with a predetermined research methodology, i.e., by using normative tests. They can serve not only to determine the mechanical parameters of a given newly emerging system but also to evaluate and compare systems existing on the market. The authors of the study not only carried out fatigue tests according to the required standard for the dental set EN14801but also, through the introduction of FEM analysis into the research, drew attention to the possibility of a broader evaluation and interpretation of the obtained results. This significantly shortens prototype work, indicating areas at risk of overload (stress concentration), finding the “weakest link” in the entire set, or the influence of system assembly parameters on achieving final results.

The minimal diameter of the dental implant INTEGRA OPTIMA was 3.35 mm, and that is why it was chosen for FEA and mechanical examination. The use of implants with the smallest possible diameter is important from the perspective of bone quantity. In anatomical conditions where there is limited bone, the doctor aims to maximize the use of available bone. If there is no need for bone reconstruction, implants with small diameters, up to 3.5 mm, are desirable.

Researchers often predetermine a specific number of cycles for these tests, typically 2, 5, or 10 million cycles. However, this predetermined cycle count is sometimes inaccurately referred to as the material’s fatigue limit for an infinite number of cycles [12,13]. To put this into perspective, considering that an individual typically has three 15 min meals per day, with each chewing cycle completed at a frequency of 1 Hz, this results in roughly 2700 mastications daily. Hence, approximately one million cycles of chewing sequences equate to a year’s worth of masticatory function [16]. In dentistry, many studies use 5 million cycles, which is roughly equivalent to five years of masticatory activity, as a benchmark to determine the fatigue limit [17,18,19,20,21].

Although this approach provides a numerical reference, it is crucial to recognize that such a straightforward mathematical correlation does not fully capture the complexities of masticatory function. However, using 5 million cycles as a reference point is valuable, particularly for comparing different systems in dental studies. This simplification helps standardize testing and provides a framework for evaluating and comparing the durability of dental materials and components under simulated masticatory conditions.

From the analysis of the results of the studies conducted on the numerical model, it was possible to hypothesize that, assuming the sought-after force value corresponding to unlimited fatigue strength would be between F = 126–183 N, this was confirmed to some extent by the results of the fatigue test [F ≈ 200–210 N], the fatigue mechanism of the dental system would primarily be influenced by the following:

- The area of the upper part of the hexagonal-cylindrical peg of the prosthetic connector.
- The area of the thread of the screw connecting the prosthetic connector to the endosteal implant.

The possible scenario that could arise as a result of cyclic loading would be that the pre-tension of the screw would cause its local plastic deformation, which consequently, before the applied cyclic loading, would “loosen” the assembly leading to the reduction in internal stresses in the screw. Such a situation would consequently affect the state of loading of the prosthetic connector, in which one could observe the first signs of the mechanism of initiation and propagation of fatigue cracks in the material.

The results indicated a good correlation between the FEM analysis and the fatigue test. They pointed to the same stressed area [the upper part of the insert, the corner of the hexagonal prosthetic connector] as the site of initiation and propagation of fatigue cracks. Additionally, the FEM analysis revealed the influence of the preload stress of the screw connecting the system. On the one hand, it ensures the system’s tightness, while on the other hand, its excessive value could potentially contribute to the loosening of the system and the emergence of “micro-movements” in the implant socket, causing its overload. One should ask why potential sources of fatigue cracks were not revealed in the screw. The explanation for this phenomenon can be found in the sequence of progressing events: microplasticity of the screw [within the thread] loosened the system, causing micro-movements in the socket, which in turn caused the formation of fatigue crack sources in the corners, partly due to large deformation resulting from the nature of the implant’s work in the test [bending]. The resulting fatigue cracks caused a change in the cross-sectional area at the loaded site, which could cause a sudden increase in stress in the screw and its plastic destruction. In the FEM analysis, it was also visible that the very mounting of the implant in the holder affects the occurring stress concentrations in the thread. If we are aware of the adopted simplifications, i.e., the way theimplantis mounteddiffers from the way it is mounted in the bone, we can dismiss its significant impact on the obtained results. In the discussed case, despite the high “sensitivity” of the implant to loading [obtained in the FEM analysis], it was still interpreted as negligible behavior in the tested system. This was reflected in the conducted test, where fatigue fractures were located in the prosthetic connector. At this point, it should be mentioned that such a conclusion was possible only due to correlating FEM results with the test on a real object. Another surprising phenomenon was the significant impact of the system’s positioning/placement, the socket according to the plane of system loading. This impact was especially visible in the hexagonal-shaped socket. The normative test does not consider this problem. However, as a consequence, it may contribute to the scale of dispersion of the obtained results. On the other hand, it seems that a round socket shape, for example, would not be burdened with such behavior. What should be investigated in the future is the influence of mounting and the different stiffness of the applied support on the research results. We already know that this can have a significant impact on the system’s survival. The differences obtained in the FEM analysis and the normative test in estimating the force corresponding to the system’s fatigue strength testify to this. Most likely, the closer the stiffness of the support mounting is to the real parameters of the patient’s bone, the higher the system’s survival rate, and we will obtain a higher value of the force corresponding to the fatigue strength. In the applied standard, we only have a general condition stated, which considers that the mounting stiffness should not be less than 3GPa. This may be insufficient for a correct interpretation of the results, knowing that the patient’s bone has varying stiffness, and it is difficult to estimate it in such a simple way.

### 4.2. Clinical Application of Narrow Implants—Survival, Success, and Marginal Bone Level under Functional Loading

Implants known as “narrow”, according to the literature, have the following diameters [mm]: 1.8, 2, 2.2, 2.4, 2.5, 3.0, 3.3, 3.5, and 3.75 [21,22,23,24,25,26,27,28,29,30,31,32,33,34,35,36,37]. Marc et al. [38] suggested three categories of division of narrow implants:

Category 1: The average functional follow-up duration for the examined dental implants measuring <3.0 mm (mini-implants) varied from 12 to 96 months. The majority of these implants were one-piece with diameters of 1.8, 2.4, or 2.5 mm. The specific indications were limited to the edentulous arch and the nonloaded frontal region. Among the studies that detailed the type of flap used, an open procedure was employed in five out of seven cases. In most instances, immediate loading with an overdenture was applied. Reported survival rates for dental implants <3.0 mm ranged from 90.9% to 100%, with only one study providing an implant success rate of 92.9%. Radiological assessments at 24 months postimplant insertion revealed an average peri-implant bone loss of 0.98 ± 0.36 mm.

Category 2: For dental implants with diameters between 3.0 and 3.25 mm, the mean follow-up duration ranged from 12 to 63 months. The prevailing study design was a single-arm prospective or retrospective study. Most of the implants in this category were two-piece, with a diameter of 3.0 mm. The primary indication for these implants was a narrow tooth gap without loading in the frontal region. The shortest implant length was 10 mm, and in each study, a flap was raised during implant insertion. Implants were either loaded directly or after a healing period of 6 to 24 weeks. Survival rates for these implants varied between 93.8% and 100%, with only one study reporting an implant success rate. The average peri-implant bone loss after 12 months was 0.78 ± 0.48 mm.

Category 3: Literature research revealed a follow-up of dental implants in category 3 (3.3 to 3.5 mm) spanning 12 to 144 months. All implants in this category were two-piece with a minimum length of 8 mm. Indications were not consistently defined but included the load-bearing posterior region in some cases. A flap was raised for implant insertion in every study, with healing occurring either sub- or transgingivally. Healing time ranged from 6 to 24 weeks. Survival rates were reported between 88.9% and 100%, and success rates ranged from 91.4% to 97.6%. Radiological assessments indicated an average peri-implant bone loss of 0.31 ± 0.03 mm after 12 months.

Changes in marginal bone level, the thickness of buccal bone, clinical results, aesthetic outcomes, and patient satisfaction are similar between immediate implant placement combined with bone augmentation in postextraction sockets with buccal bony defects of ≥5 mm and delayed implant placement after ridge preservation in the esthetic zone [39]. It demonstrates the utility of narrow dental implants, enabling patients to avoid extensive and invasive bone reconstruction procedures in the mandible or maxilla; thus, narrow implants have been thoroughly investigated in this article.

## 5. Conclusions

1. The comparison of results presented on the Wöhler curve prepared for selected systems suggests that the unlimited strength for the Optima 3.35 system is indicated at a load force level of F = 200–210 N.
2. Comparing the results of the fatigue test with the qualitative FEA analysis, it can be concluded that the value of unlimited fatigue strength at a load force level of F = 200–210 N is consistent with the qualitative results of numerical calculations where the load force was estimated at F = 180 N. The differences that were revealed probably result from the change in the pre-tension of the connecting screw during the assembly of the system as well as the method of mounting the implant with the base.
3. Observation of the prosthetic connector in the OPTIMA 3.35 system, cyclically loaded with a force of F ≈ 300 N in the area of the upper hexagonal peg, revealed a fatigue fracture consisting of a perifocal zone, primary displacements, and fatigue lines. Fatigue foci are located on both opposite sides (corners) of the hexagonal peg.
4. From the comparison of the numerical analysis with the SEM image of the fatigue fracture in the area of the upper part of the hexagonal peg of the prosthetic connector for the OPTIMA 3.35 system, satisfactory convergence of results was obtained. The areas indicated in the numerical analysis confirm that they are potential sites for the development of cracks and propagation of fatigue foci in the analyzed system.
5. The observed change in stress peak in the dental connector for two different force application surfaces shows that the positioning of the dental system (setting of the socket in relation to the force action plane) is significant in estimating the limited fatigue strength. This difference can be seen in the Wöhler curve characteristics by observing the load level for a force close to F ≈ 300 N, where significant differences in the life cycle count of the tested structure were obtained.
6. The method of modeling, correlated with fatigue testing, not only explains the impact of the analyzed geometry of the system on the test results but also explains phenomena occurring during loading, which have a significant impact on the estimation of the unlimited fatigue strength of the system.

## Figures and Tables

**Table 1:** Material of the dental set: endosteal implant + connector screw + abutment—actual material data according to the manufacturer’s certificate (titanium alloy grade 4).

Density	465	kg/m^3^
Young’s Modulus	110.000	MPa
Poisson’s Ratio	0.34	-
Yield Strength	848	MPa
Tangent Modulus	30.000	MPa

**Table 2:** Research results: reduced von Mises stresses—peak/pulse of von Mises stresses.

Area of force application	X-Z	X-Y	X-Z	X-Y
Temporal point of analysis	0.08 [s]	0.08 [s]	0.1 [s]	0.1 [s]
Value of the peak of reduced von Mises stresses in the endosteal implant	747.27 [MPa]	704.84 [MPa]	854.54 [MPa]	810.97 [MPa]
	6%		5.3%	
Value of the peak of reduced von Mises stresses in the connector screw	897.43 [MPa]	909.45 [MPa]	916.37 [MPa]	934.18 [MPa]
	1.3%		1.9%	
Value of the peak of reduced von Mises stresses in the abutment	795.85 [MPa]	831.07 [MPa]	795.68 [MPa]	839.50 [MPa]
	4.4%		5.5%	

**Table 3:** Load level and forces with life span in cycles for the OPTIMA 3. 35 dental system (material: grade 4 alloy).

Force F [N]	Standard Deviation ± [N]	Number of Cycles [n]
195,100	5525	5,000,000
198,300	5625	5,000,000
198,400	5183	5,000,000
206,600	6769	5,000,000
218,000	4853	146,546
228,200	4846	93,242
228,200	4336	66,917
248,100	4169	58,217
297,000	5835	8010
297,700	4146	21,362
297,500	4711	14,276
297,700	4313	20,461

## Data Availability

The data presented in this study are available on request from the corresponding author. The data are not publicly available due to company secret.
